# Multiple myeloma hinders erythropoiesis and causes anaemia owing to high levels of CCL3 in the bone marrow microenvironment

**DOI:** 10.1038/s41598-020-77450-y

**Published:** 2020-11-25

**Authors:** Lanting Liu, Zhen Yu, Hui Cheng, Xuehan Mao, Weiwei Sui, Shuhui Deng, Xiaojing Wei, Junqiang Lv, Chenxing Du, Jie Xu, Wenyang Huang, Shuang Xia, Gang An, Wen Zhou, Xiaoke Ma, Tao Cheng, Lugui Qiu, Mu Hao

**Affiliations:** 1 State Key Laboratory of Experimental Hematology, National Clinical Research Center for Blood Diseases, Institute of Hematology and Blood Diseases Hospital, Chinese Academy of Medical Sciences and Peking Union Medical College, Tianjin, 300020 China; 2 Department of Immunology, Key Laboratory of Immune Microenvironment and Disease of the Educational Ministry of China, Tianjin Key Laboratory of Cellular and Molecular Immunology, School of Basic Medical Sciences, Tianjin Medical University, Tianjin, 300070 China; 3 Department of Radiology, Tianjin First Central Hospital, Tianjin, 300192 China; 4 Cancer Research Institute, Key Laboratory of Carcinogenesis and Cancer Invasion, Ministry of Education; Key Laboratory of Carcinogenesis, National Health and Family Planning Commission, Central South University, Hunan, China; 5 School of Computer Science and Technology, Xidian University, Xi’an, China

**Keywords:** Myeloma, Cancer microenvironment, Anaemia, Erythropoiesis

## Abstract

Anaemia is the most common complication of myeloma and is associated with worse clinical outcomes. Although marrow replacement with myeloma cells is widely considered a mechanistic rationale for anaemia, the exact process has not been fully understood. Our large cohort of 1363 myeloma patients had more than 50% of patients with moderate or severe anaemia at the time of diagnosis. Anaemia positively correlated with myeloma cell infiltration in the bone marrow (BM) and worse patient outcomes. The quantity and erythroid differentiation of HSPCs were affected by myeloma cell infiltration in the BM. The master regulators of erythropoiesis, GATA1 and KLF1, were obviously downregulated in myeloma HSPCs. However, the gene encoding the chemokine CCL3 showed significantly upregulated expression. Elevated CCL3 in the BM plasma of myeloma further inhibited the erythropoiesis of HSPCs via activation of CCL3/CCR1/p38 signalling and suppressed GATA1 expression. Treatment with a CCR1 antagonist effectively recovered GATA1 expression and rescued erythropoiesis in HSPCs. Myeloma cell infiltration causes elevated expression of CCL3 in BM, which suppresses the erythropoiesis of HSPCs and results in anaemia by downregulating the level of GATA1 in HSPCs. Thus, our study indicates that targeting CCL3 would be a potential strategy against anaemia and improve the survival of myeloma patients.

## Introduction

Multiple myeloma (MM) is characterized by bone destruction, anaemia, and renal and immunological impairment. These complications may lead to a severe reduction in the quality of life of myeloma patients and may shorten their life expectancy^1,2^. Anaemia is the most common complication of myeloma patients at diagnosis and in almost all patients with uncontrolled disease. Given the known adverse impact of multiple myeloma on physical functioning and quality-of-life variables, including fatigue and cognitive function, managing anaemia should be an integral part of myeloma patient care. Elucidating the mechanism underlying anaemia and developing an effective treatment are critical for improving the quality of life of MM patients.

The most frequent underlying pathophysiological conditions reported previously in myeloma-related anaemia are aberrant iron metabolism, renal impairment and anaemia of chronic disease^3–5^. In patients who achieve complete remission after chemotherapy, their anaemia usually normalizes. Non-responders and relapsed myeloma patients often continue to suffer from anaemia^6^. This means that myeloma cell infiltration is highly involved in the pathogenesis of anaemia in myeloma patients. However, the mechanism of anaemia in myeloma is not fully understood.

Bone marrow is the main location where the growth and differentiation of myeloma cells and haematopoietic stem and progenitor cells (HSPCs)^7,8^. Specialized bone marrow niches control the homeostasis and differentiation of HSPCs. An aberrant microenvironment comprising myeloma cell infiltration and increased concentrations of soluble cytokines, growth factors derived from malignant cells and innocent bystander cells is beneficial for myeloma cell survival and plays a pivotal role in controlling HSPC differentiation^9–12^. Based on this, we focused on the effects and molecular mechanisms of myeloma cell infiltration in the bone marrow resulting in defective erythropoiesis of HSPCs and anaemia in the present study.

Here, we demonstrated that the quantity and erythroid differentiation of HSPCs were affected by myeloma cell infiltration in the bone marrow microenvironment. Most HSPCs were arrested in G0 phase of the cell cycle and exhibited reduced proliferation towards megakaryocyte-erythroid progenitors. We also found that the expression of the transcription factors GATA1 and KLF1 was significantly downregulated in the HSPCs of myeloma patients, especially after the cells were co-cultured with bone marrow plasma from myeloma patients. Further analysis demonstrated that the increase in the levels of the chemokine CCL3 in bone marrow plasma effectively blocks the erythropoiesis of HSPCs via downregulation of GATA1 in HSPCs by activating the p38 signalling pathway. Inhibition of CCL3 activity with a CCR1 antagonist efficiently restored the expression of GATA1 and rescued the erythropoiesis of HSPCs. These findings suggest that the aberrant microenvironment owing to myeloma cells suppressed the differentiation of HSPCs to erythrocytes. The chemokine CCL3 caused not only lytic bone lesions but also defective erythropoiesis. Targeting CCL3 is a potential strategy against anaemia and bone disease, which means killing two birds with one stone in patients with multiple myeloma.

## Results

### Anaemia is a striking symptom that predicts worse outcome in myeloma patients

**Figure 1:**
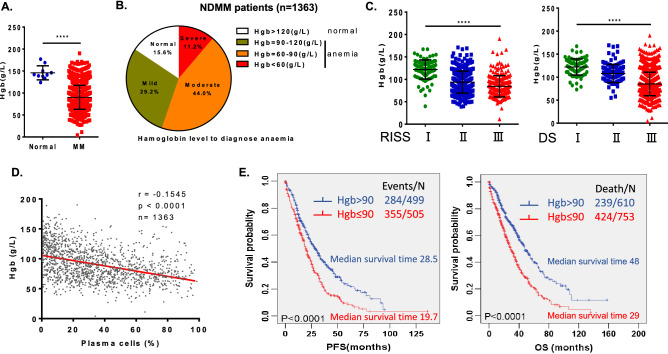
Anaemia is an important symptom of myeloma and correlates with worse outcomes in myeloma patients. (**A**) The clinical data of a large cohort of myeloma patients showed the levels of haemoglobin in normal donors (n = 10) and NDMM patients (n = 1363). (**B**) The clinical analysis showed that 84.4% (1150/1363) of NDMM patients had anaemia at diagnosis, and 55.2% (753/1363) of patients had moderate (90–120 g/L) or severe (60–90 g/L) anaemia. (**C**) The decrease in haemoglobin in NDMM patients was positively correlated with the disease stage. R-ISS stage (left) or DS stage (right). (**D**) The levels of haemoglobin in NDMM patients were negatively correlated with myeloma cell infiltration in the bone marrow (r = − 0.1545, *p* < 0.0001, n = 1363). (**E**) Kaplan–Meier analysis showed shorter survival of myeloma patients with decreased haemoglobin levels.

Newly diagnosed MM (NDMM) patients (n = 1363) were enrolled in this analysis. The patients’ clinical features are described in Table 1. Our data demonstrated that 84.4% of patients (1150/1363) had anaemia (Hgb < 120 g/L) at the time of diagnosis regardless of sex, and 55.2% (753/1363) of patients had moderate (90–120 g/L) or severe anaemia (60–90 g/L) (Fig. 1A,B) at the time of diagnosis. We also found that the reduction in Hgb was positively correlated with myeloma cell burden. The patients with advanced stage disease had lower levels of Hgb both in the DS (Durie–Salmon) staging system (80.1 ± 0.8 g/L in DS stage III) and the Revised International Staging System (RISS) (84.3 ± 1.6 g/L in R-ISS stage III; Table 1 and Fig. 1C). In particular, the level of Hgb in myeloma patients was negatively correlated with the infiltration of myeloma cells in the bone marrow (r = − 0.1545, *p* < 0.0001; Fig. 1D). Moreover, patients with anaemia had more severe bone disease, as indicated in Table 1. Kaplan–Meier analysis indicated that the survival of patients with lower levels of Hgb (n = 753, Hgb ≤ 90 g/L) was shorter than that of patients with higher levels of Hgb (n = 610, Hgb > 90 g/L). Progression-free survival (PFS) was 19.7 vs. 28.5 months (*p* < 0.0001), and the overall survival (OS) was 29.0 vs. 48.0 months (*p* < 0.0001) between the low Hgb group and high Hgb group, respectively (Fig. 1E). If the cut-off Hgb level was 60 g/L, the survival of patients with severely decreasing Hgb levels (≤ 60 g/L) was further shortened compared to that of patients with Hgb levels greater than 60 g/L (Supplementary Figure 2). Our findings clearly demonstrated that anaemia is a striking symptom and efficiently predicted worse outcomes of patients with myeloma. Specifically, Hgb levels were negatively correlated with the infiltration of myeloma cells.

**Table 1:** Baseline characteristics of 1363 NDMM patients.

Characteristics	All patients (%)	Hgb > 120 g/L (%)	Hgb ≤ 120 g/L (%)	*p* value
No. of patients	1363	213 (15.6%)	1150 (84.4%)	
**Gender**				
Male	839(61.6%)	166(77.9%)	673 (58.5%)	
Female	524 (38.4%)	47 (22.1%)	477 (41.5%)	
**Age**				< 0.001
< 65 year	1016(74.5%)	178(83.6%)	838(72.9%)	
≥ 65 year	347 (25.5%)	35 (16.4%)	312(27.1%)	
**Ig isotype**				
IgG	654 (48.5%)	94(47.0%)	560 (48.7%)	
IgA	317 (23.5%)	52 (26.0%)	265 (23.1%)	
IgD	65 (4.8%)	8 (4.0%)	57 (5.0%)	
Light chain	272 (20.2%)	38 (19.0%)	234 (20.4%)	
Non-secretory	41 (3.0%)	8 (4.0%)	33 (2.8%)	
**RISS stage**				< 0.0001
I	84 (13.5%)	49 (40.2%)	35 (6.3%)	
II	389 (55.8%)	56 (45.9%)	333 (59.7%)	
III	207 (30.7%)	17 (13.9%)	190 (34.0%)	
**DS stage**				< 0.0001
I	86 (6.5%)	50 (25.1%)	36 (3.2%)	
II	122 (9.2%)	29 (14.6%)	93 (8.3%)	
III	1117(84.3%)	120 (60.3%)	997(88.5%)	
BM-infiltration (%) median; range	29.5 (0–98.5)	9 (0–91.5)	33 (0–98.5)	< 0.0001
**MBD**				0.014
0–2	47 (31.5%)	12 (60.0%)	35 (27.1%)	
3–4	102 (68.5%)	8 (40.0%)	94 (72.9%)	
Hemoglobin (g/L) median; range	89 (4–197)	132(121–197)	81(4–120)	

*RISS* Revised international staging system, *DS* Durie Salmon, *MBD* myeloma bone disease.

### Myeloma bone marrow suppresses the growth and differentiation of HSPCs

**Figure 2:**
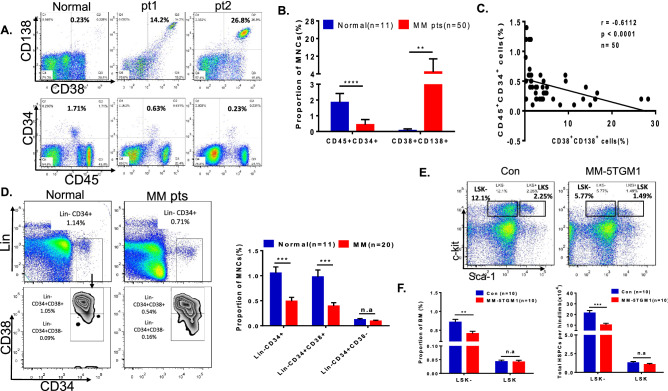
The proportion of HPCs is significantly decreased in the myeloma bone marrow in both primary patients and the MM mouse model. (**A**) Flow cytometry was used to detect CD138^+^CD38^+^ myeloma cells and CD45^+^CD34^+^ HSPCs in bone marrow samples from NDMM patients and normal donors. (**B**) Bar charts represent the mean proportions of CD138^+^CD38^+^ plasma cells and CD45^+^CD34^+^ HSPCs in normal donors and NDMM patients. (**C**) The proportion of CD45^+^CD34^+^ cells in NDMM patients was negatively correlated with the proportion of CD138^+^ myeloma cells (r =  − 0.6112, *p* < 0.0001, n = 50). (**D**) Flow plots (left) and a histogram (right) show the percentage of HSPCs in normal donors and NDMM patients. The subset composition of HSPCs (Lin^−^CD34^+^), including HPCs (Lin^−^CD34^+^CD38^+^) and HSCs (Lin^−^CD34^+^CD38^-^), was determined in BM samples from normal donors and NDMM patients. (**E**) The subsets of HSPCs, including HPCs (Lin^−^, c-kit^+^, Sca-1^-^) and HSCs (Lin^−^, c-kit^+^, Sca-1^+^), were determined in BM samples from the 5TGM1 myeloma mouse model and control mice. (**F**) The proportion (left) and absolute number (right) of HSPC subsets were investigated (n = 10 per group).

Flow cytometry analysis revealed that the population of CD45^+^CD34^+^ HSPCs was significantly decreased in bone marrow biopsy samples of myeloma patients compared to the healthy controls (0.43% ± 0.04% vs. 1.84% ± 0.17%, *p* < 0.0001; Fig. 2A,B). The number of CD45^+^CD34^+^ HSPCs was negatively correlated with the number of CD38^+^CD138^+^ myeloma cells in the bone marrow of MM patients (r = − 0.6112, *p* < 0.0001; Fig. 2C). As Fig. 2D shows, the number of Lin^-^CD34^+^CD38^+^ HPCs was dramatically depressed in the bone marrow of myeloma patients compared with that of healthy patients (0.39% ± 0.07% vs. 0.98% ± 0.14%, *p* = 0.0002). However, the number of Lin^−^CD34^+^CD38^−^ HSCs did not show an obvious decrease between the two groups (0.09% ± 0.02% vs. 0.12% ± 0.02%). The data indicated that the decrease in HSPCs (Lin^−^CD34^+^) was mainly due to the differentiation defects of HSCs, which caused a decrease in the HPC (haematopoietic progenitor cells, Lin^-^CD34^+^CD38^+^) population but not the HSC (haematopoietic stem cells, Lin^-^CD34^+^CD38^−^) population.

To confirm these results, the C57BL/KaLwRij murine myeloma model was utilized. Six weeks after injection of the 5TGM1 mouse myeloma cell line (2 × 10^6^/mouse), the elevated CD138^+^ mouse myeloma cell population (10.9% ± 1.2%, n = 6) and IgG2b levels (451 ± 10 µg/mL, n = 6) in the bone marrow of MM-5TGM1 mice indicated the successful proliferation of myeloma cells (Supplementary Figure 3A&3B). Routine tests of peripheral blood revealed a significant decrease in Hgb levels in all mice injected with MM-5TGM1 myeloma cells compared those in tumour-free control mice (119 ± 5 vs. 142 ± 5 g/L, n = 10; Supplementary Figure 3C. In this myeloma mouse model, we also did not find significant variation in either the proportion or absolute number of haematopoietic stem cells (LSK: Lin^−^c-Kit^+^Sca-1^+^) (0.042% ± 0.006% vs. 0.043% ± 0.005%; Fig. 2E,F). However, the frequency and absolute number of myeloid progenitor cells (LSK^−^: Lin^−^c-Kit^+^Sca1^−^) in the bone marrow of MM-5TGM1 mice were significantly reduced compared to those in tumour-free control mice. In these two mouse groups, the frequency of LSK^−^ cells was (0.41% ± 0.06%) versus (0.71% ± 0.07%), *p* = 0.0052, and the absolute number was (10.27 ± 1.50) × 10^4^ versus (21.43 ± 2.23) × 10^4^, *p* = 0.0006, respectively. The results of the myeloma patient and mouse model experiments showed that myeloma cell infiltration mainly caused a decrease in HPCs in the bone marrow. These data also indicated that the differentiation of HSPCs was inhibited in the microenvironment of myeloma.

### The differentiation of HSPCs is arrested in the HSC stage in the myeloma bone marrow

**Figure 3:**
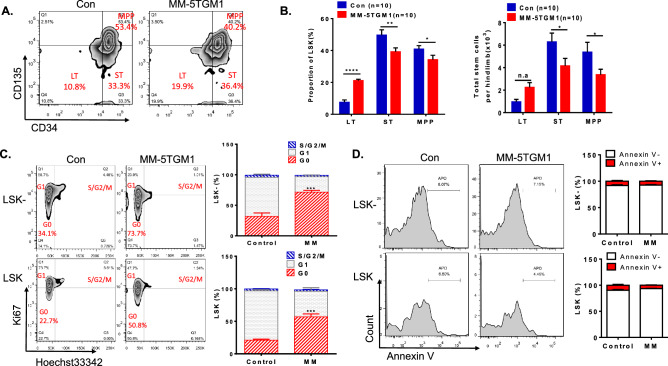
The differentiation of HSPCs arrested in the HSC stage in the myeloma bone marrow. (**A**) The subset of LSK cells, including LT-HSCs (Lin^−^, c-kit^+^, Sca-1^+^, CD34^-^, CD135^−^), ST-HSCs (Lin^−^, c-kit^+^, Sca-1^+^, CD34^+^, CD135^−^), and MPPs (Lin^−^, c-kit^+^, Sca-1^+^, CD34^+^, CD135^+^), was determined in BM samples. (**B**) The proportions and absolute numbers of the LSK subset are shown. (**C**) Representative flow graphs (left) and histograms (right) show the cell cycle status of LSK^−^ (Lin^−^c^−^Kit^+^Sca-1^-^) and LSK (Lin^−^c^−^Kit^+^Sca-1^+^) cells in BM from MM-5TGM1 mice and tumour-free control mice (n = 6 in each group). (**D**) Apoptosis of LSK^-^ and LSK cells from MM-5TGM1 mice and tumour-free control mice is shown (n = 6 per group).

Long-term repopulating (LT) haematopoietic stem cells (HSCs) are the progenitor cells at the top of the haematopoietic hierarchy. Our data showed that the frequency of LT-HSCs (Lin^−^, c-kit^+^, Sca-1^+^, CD34^−^, CD135^-^) in the bone marrow of the myeloma mouse model was notably increased compared to that in control mice (21.13% ± 0.85% vs. 7.55% ± 1.46%, *p* < 0.0001; Fig. 3A,B). However, the frequencies of short-term HSCs (ST-HSCs: Lin^-^, c-kit^+^, Sca-1^+^, CD34^+^, CD135^−^) and multipotent progenitor MPPs (Lin^−^, c-kit^+^, Sca-1^+^, CD34^+^, CD135^+^) decreased significantly. Similarly, the absolute number of ST-HSCs and MPPs progressively decreased; by contrast, the absolute LT-HSC count showed no obvious variation. The results further suggested that the differentiation of LT-HSCs was defective in the bone marrow of myeloma mice.

Further analysis of the cell cycle and apoptosis showed that most HSPCs (LSK: Lin^−^c^-^Kit^+^Sca-1^+^, and LSK^−^: Lin^−^c^-^Kit^+^Sca-1^-^) were arrested in G0 phase of the cell cycle in the myeloma model mice compared with tumour-free control mice (G0 phase, LSK^−^: 41.88% ± 6.97% vs. 18.48% ± 1.56%, *p* = 0.0083; LSK: 55.60% ± 2.61% vs. 18.85% ± 1.01%, *p* < 0.0001; Fig. 3C). However, no obvious apoptosis was detected in HSPCs from myeloma mice (LSK^−^: 8.24% ± 1.48% vs. 7.30% ± 0.85%; LSK: 9.71% ± 1.53% vs. 6.60% ± 0.52%; Fig. 3D). Therefore, our results demonstrated that cell cycle arrest, but not apoptosis, likely affected the differentiation of HSPCs in response to the myeloma microenvironment.

### Erythrocyte differentiation of HSPCs is impaired in the myeloma microenvironment

**Figure 4:**
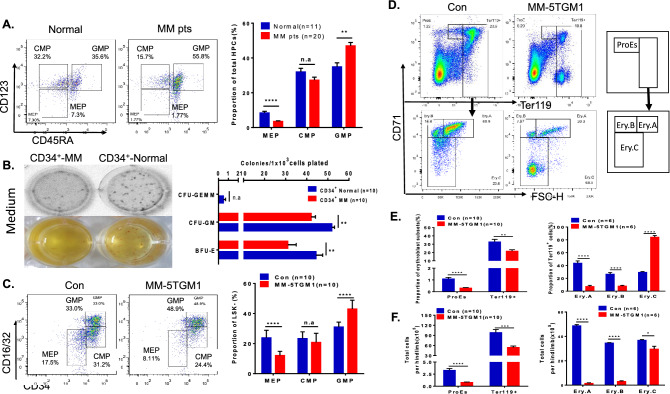
Erythroid differentiation of HSPCs was inhibited under the bone marrow microenvironment of MM patients and the 5TGM1 MM mouse model. (**A**) Flow plots (left) and a histogram (right) show the percentage of HSPCs in normal donors (n = 11) and NDMM patients (n = 20). The subset composition of HSPCs, including CMP (Lin^−^, CD34^+^, CD38^+^, CD123^lo^, CD45RA^−^), GMP (Lin^−^, CD34^+^, CD38^+^, CD123^lo^, CD45RA^+^), and MEP (Lin^−^, CD34^+^, CD38^+^, CD123^−^, CD45RA^−^), was determined. (**B**) Equal numbers of CD34^+^ cells from the BM of normal donors and NDMM patients were seeded in semisolid methylcellulose complete medium. The numbers of BFU-E, CFU-GM and CFU-GEMM colonies were calculated after 14 days of co-culture (upper). The means of the absolute numbers of colonies are shown (lower, n = 10 per group). (**C**) The subset composition of HSPCs, including CMP (Lin^−^, c-kit^+^, Sca-1^−^, CD34^+^, CD16/32^lo^), GMP (Lin^−^, c-kit^+^, Sca-1^−^, CD34^+^, CD16/32^hi^), and MEP (Lin^−^, c-kit^+^, Sca-1^−^, CD34^−^, CD16/32^−^), was determined in BM samples from MM-5TGM1 mice and control mice. (**D**) Erythroblast subsets were analysed by flow cytometry based on the expression of CD71 and Ter-119 as follows: ProE (CD71^+^ Ter-119^low^), Ery.A, Ery.B, and Ery.C (Ter-119^+^). (**E**) The proportion of different erythroblasts in BM from MM-5TGM1 mice and tumour-free control mice. (**F**) Absolute number of ProEs, Ter-119^+^, Ery.A, Ery.B and Ery.C in the BM of MM-5TGM1 mice and tumour-free control mice.

Megakaryocytic-erythroid progenitors (MEPs) are considered to be the classical progenitors of functional red blood cells and platelets^13^. We analysed the quantity of common myeloid progenitors (CMPs) and their progeny, granulocyte-monocyte progenitors (GMPs) and megakaryocyte-erythrocyte progenitors (MEPs) in the bone marrow of patients with myeloma. Our results indicated that the population of MEPs (Lin^-^CD34^+^CD38^+^CD45RA^−^CD123^−^) was the most significantly suppressed in myeloma patients (n = 20), as shown in Fig. 4A (3.52% ± 0.35% vs. 8.43% ± 0.89%, *p* < 0.0001). Furthermore, a colony formation assay of CD34^+^ HSPCs from myeloma patients or healthy control subjects was performed. The data indicated that colony formation, especially BFU-E colony formation, was significantly defective in myeloma CD34^+^ HSPCs compared to normal control CD34^+^ HSPCs (31.00 ± 3.98 vs. 44.40 ± 3.14, *p* < 0.01, Fig. 4B). Experiments in the myeloma mouse model confirmed these results. The proportion of MEPs was significantly decreased in MM-5TGM1 mice compared to tumour-free control mice (12.10% ± 0.87% vs. 23.85% ± 1.59%, *p* < 0.0001, Fig. 4C). Moreover, the quantities of the pro-erythroblast (ProEs: CD71^+^Ter119^low^) and Ter119^+^ erythroblast subpopulations (Ery.A, Ery.B and Ery.C) were notably reduced in MM-5TGM1 mice (ProEs: (0.78 ± 0.10) × 10^5^ vs. (3.28 ± 0.38) × 10^5^, *p* < 0.0001; Ter119^+^: (54.10 ± 4.25) × 10^5^ vs. (98.25 ± 9.20) × 10^5^, *p* < 0.001; Fig. 4D–F). However, the proportion of Ter119^+^71^−^ (Ery.C) increased notably. In order to confirm this result, we further analyzed the bone marrow smears of myeloma patients (n = 10) and healthy donors (n = 4). We found that the proportion of erythroblasts decreased in bone marrow of MM patients (supplementary Figure 4A), but the proportion of late erythroblast cells increased in erythroblasts subset (supplementary Figure 4B).

### RNA sequencing analysis indicates the downregulation of GATA1 and KLF1 and upregulation of CCL3 in CD34^+^ cells from myeloma patients

**Figure 5:**
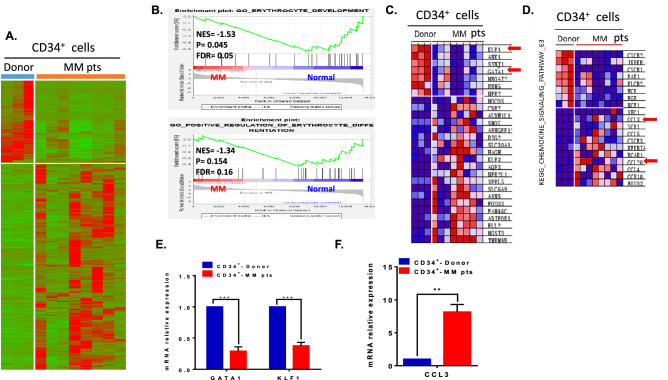
RNA-seq identified the downregulation of transcription factors involved in erythroid differentiation of HSPCs in the myeloma microenvironment. (**A**) Heatmap of differentially expressed genes in CD34^+^ cells from MM patients and healthy controls. (**B**) GSEA comparison of gene expression inCD34^+^ cells from MM patients and normal controls with respect to erythrocyte development (upper panel) and positive regulation of erythrocyte differentiation (lower panel). The normalized enrichment scores (NESs), *p* values and FDR q-values are indicated in each plot. (**C**) GSEA revealed that the expression of the transcription factors GATA1 and KLF1 was significantly decreased in CD34^+^ cells from MM patients. (**D**) KEGG (Kyoto Encyclopedia of Genes and Genomes) pathway enrichment analysis of the genes involved in the chemokine signalling pathway. CCL3 was upregulated in CD34^+^ cells from myeloma patients. (**E**) RT-PCR indicated that the expression of the transcription factors GATA1 and KLF1 was significantly decreased in CD34^+^ cells from MM patients. (**F**) RT-PCR indicated that the expression of CCL3 was significantly increased in CD34^+^ cells from MM patients.

To clarify the signalling pathway involved in the defective erythropoiesis of HSPCs within the bone marrow of myeloma patients, RNA sequencing was performed. A total of 1243 differentially expressed genes (DEGs) were identified in CD34^+^ cells between myeloma patients (n = 8) and healthy donors (n = 3), 852 of which were upregulated and 391 of which were downregulated (FC > 2.0, *p* < 0.01; Fig. 5A). Gene set enrichment analysis^14^ revealed that the gene sets related to erythrocyte development and erythrocyte differentiation were significantly suppressed in CD34^+^ cells from MM patients but enriched in those from normal control subjects (Fig. 5B). More importantly, the expression of transcription factors, such as GATA1 and KLF1, which govern erythrocyte differentiation, was significantly downregulated in CD34^+^ HSPCs from myeloma patients. Meanwhile, the expression of genes involved in the chemokine signalling pathway, including CCL3, CCL4, CCL5 and CCL20, was upregulated (Fig. 5C–F). This result supports the notion that cytokines or chemokines in the myeloma microenvironment play a pivotal role in the suppression of HSPC differentiation through the downregulation of transcription factor expression.

### High levels of CCL3 in myeloma bone marrow plasma cells inhibits erythrocyte differentiation

**Figure 6:**
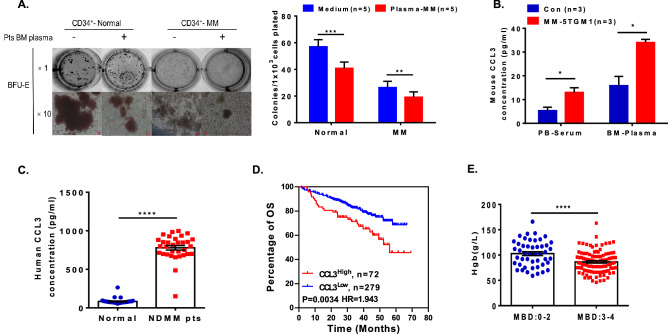
Bone marrow plasma inhibited erythroid colony formation of HSPCs by increasing CCL3 levels in the MM microenvironment. (**A**) A CFC assay was utilized to quantify the BFU-E colony formation of CD34^+^ cells after co-culture with or without BM plasma from MM patients for 14 days. The absolute numbers of BFU-E colonies are shown on the right. (**B**) The concentrations of CCL3 in the bone marrow plasma cells and PB serum of 5TGM1 MM mice and control mice were detected with ELISA. (**C**) The concentration of CCL3 in the bone marrow plasma cells of MM patients was determined with ELISA (normal, n = 40; NDMM pts, n = 32). (**D**) Kaplan–Meier analysis showed shorter overall survival in MM patients with high levels of CCL3 based on the GEO dataset GSE2658. (**E**) The clinical data indicated that the more severe the bone disease is (MBD 3–4), the greater the decrease in haemoglobin in MM patients (MBD: 0–2, n = 47; MBD: 3–4, n = 102).

In addition to the intrinsic deficits in the erythropoiesis of CD34^+^ HSPCs with downregulated GATA1 expression in a myeloma bone marrow microenvironment, we found that the bone marrow plasma cells of myeloma patients further suppressed the BFU-E formation of CD34^+^ cells both from myeloma patients and healthy donors (Fig. 6A). Our previous clinical analysis showed in Table 1 that bone disease was correlated with anaemia in myeloma patients. CCL3 is one of the major cytokines secreted by MM cells that promotes tumour cell proliferation and disrupts normal haematopoietic niches, thus inducing myeloma-related bone disease^15,16^. Our RNA-seq data indicated that the expression levels of the chemokines CCL3 and CCL20, which play an important role in the pathogenesis of myeloma-induced bone disease^17^, were upregulated in CD34^+^ cells in bone marrow from myeloma patients. Further ELISA analysis showed that the secreted level of CCL3 significantly increased in the bone marrow plasma cells of myeloma patients and in the MM-5TGM1 mouse model (human plasma: 779.2 ± 28.97 pg/mL vs. 83.52 ± 5.31 pg/mL, *p* < 0.0001; mouse serum: 13.03 ± 1.94 pg/mL vs. 5.33 ± 1.46 pg/mL, *p* = 0.0317; mouse plasma: 34.03 ± 1.32 pg/mL vs. 15.86 ± 3.88 pg/mL, *p* = 0.0114; Fig. 6B,C). The Kaplan–Meier analysis based on the GEO data of individuals with NDMM (GSE2658) indicated that a high level of CCL3 significantly correlated with shorter overall survival in patients with myeloma (56.05 months vs. unreached, HR = 1.943, *p* = 0.0034; Fig. 6D). In particular, the clinical characteristics of patients with anaemia indicated that the Hgb level was negatively correlated with bone disease. The patients with higher Hgb [102.9 ± 3.72 g/L (n = 47)] had mild bone disease (0–2 stage), and the patients with advanced anaemia [86.38 ± 2.13 g/L (n = 102)] had severe bone disease (3–4 stage) (*p* < 0.0001, Fig. 6E). These results further support the notion that soluble CCL3 in the myeloma microenvironment is involved in the process of myeloma-related anaemia.

### Activation of the CCL3/CCR1/p38 signalling pathway disrupts erythroid differentiation

**Figure 7:**
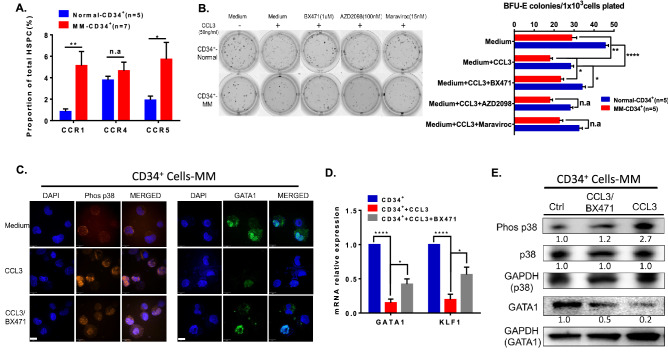
A high level of CCL3 suppresses erythroid differentiation of HSPCs via activation of the CCL3/CCR1/p38 signalling pathway in MM. (**A**) Flow cytometry analysis showing the expression of CCL3 receptors (CCR1, CCR4 and CCR5) in CD34^+^ cells from normal donors and NDMM patients. (**B**) The CFC assay showed the BFU-E colony-forming ability of CD34^+^ cells in the presence or absence of CCL3 (50 ng/ml) and the CCR1 antagonist BX471 (1 μM), the CCR4 antagonist AZD2098 (100 nM), or the CCR5 antagonist maraviroc (15 nM) (left graphs). The histograms indicate the absolute number of BFU-E colonies formed (right). (**C**) Immunofluorescence staining of phosphorylated p38 (orange) and GATA1 (green) in EPO-induced HSPC (CD34^+^) differentiation in the presence or absence of CCL3/BX471. Nuclei were stained with DAPI. Fluorescence images viewed under a confocal microscope were acquired. The scale bar indicates 10 μm. (**D**) RT-PCR analysis showed the expression levels of GATA1 and KLF1 in CD34^+^ cells in the presence or absence of CCL3/BX471. GAPDH expression was used as an internal control. (**E**) Western blotting analysis of phosphorylated p38 (phos-p38), total p38 and GATA1 after the induction of erythroid differentiation in the presence or absence of CCL3/BX471. GAPDH expression was used as an internal control.

Our previous data indicated that the high level of CCL3 in the myeloma bone marrow microenvironment is probably involved in the myeloma-mediated inhibition of erythropoiesis. Therefore, the expression of CCL3 receptors (CCR1, CCR4 and CCR5) on the surface of CD34^+^ cells was detected by flow cytometry. The data showed that the levels of CCR1 and CCR5, but not CCR4, were significantly increased on the HSPCs of myeloma patients compared to healthy donors (CCR1: 5.13% ± 1.32% vs. 0.85% ± 0.24%, *p* = 0.0096; CCR5: 5.72% ± 1.57% vs. 1.91% ± 0.37%, *p* = 0.0199; Fig. 7A). Moreover, antagonists of CCR1 (BX471), CCR4 (AZD2098) and CCR5 (Maraviroc) were used to inhibit CCL3 activity in the co-culture system of CD34^+^ cells and bone marrow plasma cells from patients. As shown in Fig. 7B, we found that blocking CCL3/CCR1 signalling with BX471 efficiently restored BFU-E formation. However, the CCR4 antagonist (AZD2098) and CCR5 antagonist (Maraviroc) had limited effects on rescuing BFU-E formation. As a downstream effector of CCL3/CCR1 signalling, phosphorylated p38 in HSPCs treated with CCL3 and the CCR1 antagonist BX471 was further analysed. Immunofluorescence staining indicated that CCL3 effectively activates p38 signalling with high levels of phosphorylated p38 in CD34^+^ cells derived from myeloma patients. Importantly, blocking the CCL3 signal with BX471 (an antagonist of CCR1) effectively restored the effect of CCL3 on the downregulation of the transcription factor GATA1 in CD34^+^ cells in the myeloma microenvironment (Fig. 7C). Real-time PCR and Western blotting further indicated the recovery of GATA1 expression at both the mRNA and protein levels after blocking the CCL3/CCR1/phos-p38 signalling pathway in CD34^+^ cells from myeloma patients (Fig. 7D,E). Compared to the CD34^+^ cells from myeloma bone marrow, the expression of GATA1 in normal CD34^+^ slightly suppressed by the CCL3 treatment which also caused by pho-p38 (supplementary Figure 5). Taken together, the results demonstrated that a high level of CCL3 activates p38 signalling, resulting in the suppression of transcription factor GATA1 expression and blocking erythroid differentiation of CD34^+^ HSPCs in the MM bone marrow microenvironment.

## Discussion

Anaemia is the most common complication of many kinds of cancer, including MM, and is associated with worse clinical outcomes, including longer length of hospital stay, diminished quality of life, and increased morbidity and mortality^18,19^. The most frequent underlying pathophysiological mechanisms reported previously in tumour-induced anaemia include aberrant iron metabolism, renal impairment, anaemia of chronic disease and a hypoxic microenvironment^20,21^. Although marrow replacement by malignant plasma cell infiltration is widely considered the basis for myeloma-related anaemia^22^, the molecular mechanism has not been fully clarified.

In this study, the data of our large cohort of 1363 newly diagnosed myeloma patients demonstrated that 84.4% of patients had anaemia regardless of sex (Hgb < 120 g/L), and more than 50% (753/1363) of patients had moderate (Hgb = 90–120 g/L) or severe anaemia (Hgb = 60–90 g/L) at the time of diagnosis. Anaemia was positively correlated with myeloma cell infiltration in the bone marrow of the patients and worsened with the progression of the disease. The patients with anaemia had significantly shorter progression-free survival and overall survival (medium PFS, 19.7 months vs. 28.5 months, *p* < 0.0001; medium OS, 29.0 months vs 48.0 months, *p* < 0.0001; Hgb cut-off 90 g/L).

Bone marrow is the main location of the growth and differentiation of hematopoietic stem cells (HSPCs). The interactions of HSPCs with other cells, secreted and membrane-bound proteins and extracellular matrix components in the bone marrow regulate HSPC differentiation^12,23^. Previously, Ingmar Bruns and colleagues reported that HSPCs were suppressed under a myeloma microenvironment^24^. Our results further demonstrated that the number of HPCs (Lin^-^CD34^+^CD38^+^), but not HSCs (Lin^-^CD34^+^CD38^−^), was more significantly decreased in the bone marrow of myeloma and negatively correlated with the quantity of CD38^+^CD138^+^ tumour cells (r =  − 0.6112). This result was confirmed in a myeloma mouse model. In particular, the LSK, short-term LSK and MPP populations, but not the long-term LSK population, were decreased in the bone marrow of myeloma mice. These data suggested that the differentiation of HPCs from HSCs was blocked under the microenvironment of myeloma. We also found that HSPCs were arrested in G0 phase and that apoptosis was not significantly changed in the bone marrow microenvironment of myeloma. Flow cytometry data showed that the population of MEPs, which differentiated from HPCs and are precursors of erythrocytes, was decreased both in the bone marrow of myeloma patients and in the MM mouse model (MM-5TGM1). We found that the frequencies of the ProEs and the A and B erythroblasts (Ery.A and Ery.B) subpopulations were reduced while that of the C erythroblasts (Ery.C) were increased in myeloma mice (Fig. 4D,E). This result was consistent with the data of MM patients shown in supplementary Figure 4. These results from patients and mouse model suggested that the differentiation of erythropoiesis was defected under the microenvironment of myeloma. However, we did not find the notable changes of Annexin V^+^ apoptosis cells and the cell cycle arrest in Ery.C (data not shown). Moreover, due to the infiltration of myeloma cells, macrophage function was abnormal^2,25^. We speculate that enucleation and differentiation of Ery.C cells were impacted under the microenvironment of myeloma with the macrophage disfunction. This would be involved in the mechanisms for the Ery.C cell accumulation. This hypothesis needs to be further investigated in the future. Our data also showed that the erythrocyte differentiation (BFU-E and CFU-GM) was further inhibited in CD34^+^ cells derived from MM patients compared with those derived from healthy donors. Therefore, these results supported the halted differentiation of HSPCs developmentally arrested in the HSC stage that is caused by MM cell infiltration.

To further clarify the molecular mechanism of the defective differentiation of HSPCs in the bone marrow of myeloma, high-throughput RNA-seq was utilized to determine the differential gene expression in CD34^+^ cells between MM patients and healthy donors. There were 1243 DEGs, of which 852 were upregulated and 391 were downregulated, in CD34^+^ cells of MM patients compared to healthy controls (*p* < 0.01). In particular, the expression of the master regulators of erythropoiesis^26–28^, including GATA1 and KLF1, was obviously decreased in myeloma CD34^+^ cells (FC <  − 2.0, *p* < 0.01). However, the expression of chemokine genes, such as CCL3 and CCL20, was significantly upregulated. The results suggested that the gene expression profile of HSPCs varied inherently under the bone marrow microenvironment of myeloma, which would disrupt erythroid differentiation.

In addition, it has been well established that malignant plasma cell infiltration disrupts bone marrow homeostasis between the highly organized cellular and extracellular compartments^9^. The release of cytokines and growth factors from malignant cells and nonneoplastic bystander cells leads to alterations in the bone marrow cytokine milieu, which interferes with the behaviour of HSPCs^29^. Our study showed that the BFU-E formation of HSPCs was further diminished when they were co-cultured with the bone marrow plasma cells of myeloma patients. Interestingly, our clinical data clearly showed that anaemia was positively correlated with bone disease severity in patients with myeloma. Patients with more severe bone lytic lesions would concurrently have more severe anaemia. These results implied that the occurrence of anaemia was highly associated with bone disease. There are some common mechanisms involved in osteoclast differentiation and erythropoiesis induced by myeloma cell infiltration. Fields SZ et al. reported that dysregulated signalling by activin receptor pathways is implicated in certain instances of anaemia, myeloma-associated osteolysis, and metastatic bone disease, as well as in carcinogenesis^30^. Deshet-Unger N and colleagues recently reported that erythropoietin (EPO), the cytokine required for erythropoiesis, directly stimulates osteoclast precursors and induces bone loss in murine multiple myeloma^31,32^. EPO targets the monocytic lineage by increasing the number of bone monocytes/macrophages, preosteoclasts, and mature osteoclasts. Their study also suggested the potential mechanism involved in both erythropoiesis and bone loss. However, the research of Bordini et al.^25^ excluded the possibility that anaemia results from defective erythropoietin production, inflammation or increased hepcidin expression in myeloma.

Cytokines and chemokines are the main components of bone marrow plasma cells and play a prominent role in the pathogenesis of myeloma-related bone disease. CCL20 is a chemokine involved in the Th17 pathway and has also been implicated in MM osteolytic disease^33^. The chemokine cytokine ligand 3 (CCL3/MIP-1α) is a pro-inflammatory protein and chemokine mainly secreted by myeloma plasma cells and contributes to the development of myeloma-induced bone disease by supporting tumour growth and regulating osteoclast (OC) differentiation. CCL3 also reduces bone formation by inhibiting osteoblast (OB) function and therefore contributes to OB/OC uncoupling in MM^15,16^. Currently, the role of CCL3 in the erythropoietic lineage is not fully understood. Chen et al. and Lentzsch et al. reported that the interaction of CCL3 and CCR1 effectively triggered the activation of MAPK and AKT pathways, which are involved in cell proliferation and differentiation^34,35^. A recent study from our colleagues Wang et al.^36^ showed that the increase in CCL3 levels in AML bone marrow partially causes defects in erythropoiesis and results in leukaemia-related anaemia. CCL3 elevation suppressed erythropoiesis in the bone marrow of AML via CCL3/CCR1/phos-p38 activation. Perturbations in the bone marrow microenvironment are increasingly recognized to have key roles in initiating and supporting oncogenesis in both AML and MM^37^. Increased CCL3 expression has also been reported in some bone marrow failure syndromes, such as aplastic anaemia^38^. Based on previous reports, we first analysed our clinical data. We found that the level of CCL3 was significantly elevated in the BM plasma cells of MM. Consistent with Lentzsch et al.^35^, myeloma patients with high levels of CCL3 had shorter survival (Fig. 6). Moreover, the expression of the CCL3 receptors CCR1 and CCR5 was significantly increased on the surface of HSPCs from myeloma patients. CCR1 and CCR5 are the key players involved in the localization and growth of myeloma cells and in the development of osteolytic lesions^39,40^. Therefore, the increased levels of CCR1 and CCR5 expression on HSPCs indicated that myeloma cells adhere to HSPCs and promote myeloma cell localization to and proliferation in bone marrow. However, this speculation needs to be further investigated in the future. Our data also showed that increasing CCL3 levels effectively inhibited the BFU-E formation of CD34^+^ HSPCs.

The MAPK signalling pathway, which includes ERK, p38 and JNK, is an important downstream modulator of CCL3 and plays a pivotal role in myeloma cell proliferation and myeloma-mediated bone disease^41^. Hernandez et al. reported that acetylation and MAPK phosphorylation cooperate to regulate the degradation of active GATA-1^42^. Therefore, we investigated the effect of CCL3/phos-p38 activation on the erythropoiesis of HSPCs in the myeloma microenvironment. Interestingly, we found that a high level of CCL3 significantly suppressed GATA1 expression at both the mRNA and protein levels in CD34^+^ cells in myeloma bone marrow. Our co-culture and rescue studies showed that a high level of CCL3 promoted the phosphorylation of p38 and suppressed the transcription and protein expression of GATA1. Inhibition of CCL3 activity with an antagonist of CCR1 (BX471) effectively restored the expression of GATA1 via inhibition of p38 phosphorylation and rescued BFU-E colony formation. Altogether, the data from our study showed that CCL3/CCR1/phos-p38 plays a pivotal role in erythropoiesis of myeloma HSPCs via downregulation of the transcription factor GATA1.

In summary, our study demonstrated that the MM microenvironment suppresses the erythroid differentiation capability of HSPCs and resulted in myeloma-related anaemia. Elevated CCL3 levels repress the erythroid differentiation of HSPCs in the bone marrow niche by suppressing the expression of the core erythroid transcription factors GATA1 and KLF1, causing insufficient erythropoiesis, as shown in our working model (Supplementary Figure 6). CCL3 is an important chemokine in myeloma pathogenesis that not only participates in inflammatory reactions and bone disease^40^ and is an adjuvant for immunotherapy^43,44^ but also plays a suppressive role in erythropoiesis and anaemia, as we reported here. Therefore, our study strongly supports the rationale for the development of novel agents that simultaneously suppress myeloma cell growth and myeloma-related bone disease and compensate for normal haematopoiesis to prevent the activation of CCL3 and consequently improve myeloma-related anaemia.

## Conclusions

Our results demonstrated that myeloma cells secreted high levels of CCL3 in the bone marrow microenvironment and caused defective erythropoiesis of HSPCs by downregulating the expression of transcription factors in HSPCs, which resulted in anaemia. Targeting CCL3 would be a potential treatment strategy against bone disease and anaemia, which could essentially “kill two birds with one stone” in patients with multiple myeloma.

## Methods

### Clinical data analysis

A total of 1363 newly diagnosed MM patients who visited our centre from 1997 to 2017 were enrolled in this analysis. The clinical features are described in Table 1. Multiple myeloma and related anaemia were diagnosed according to the criteria of the International Myeloma Working Group and the World Health Organization^45,46^.

### Patient samples

Bone marrow specimens from 50 newly diagnosed MM patients and 11 healthy control subjects were used in the in vitro study. Table 2 shows the detailed characteristics of the patients. Bone marrow biopsies were obtained following the protocol of the Department of Lymphoma and Myeloma, Institute of Hematology & Blood Disease Hospital, Chinese Academy of Medical Sciences and Peking Union Medical College (Tianjin, China). All subjects involved in the study provided written informed consent according to the Declaration of Helsinki; this research was approved by the Medical Ethics Committee of the Chinese Academy of Medical Sciences, Institute of Hematology and Blood Diseases Hospital Ethics Board (KT2019022-EC-1, 2019-03-08).

**Table 2:** Baseline characteristics of 50 NDMM patients.

Characteristics	All patients (%)	Hgb > 90 g/L (%)	Hgb ≤ 90 g/L (%)	*p*
No. of patients	50	24 (48.0%)	26 (52.0%)	
**Sex**				
Male	29 (58%)	11 (57.1%)	9(58.1%)	
Female	21 (42%)	13 (42.9%)	17(41.9%)	
Age/median; range	56 (33–68)	53 (36–68)	56 (33–68)	0.654
BM-infiltration (%) median; range	60 (5–90)	30 (5–85)	65 (8–90)	< 0.01
**Ig isotype**				
IgG	23 (46.0%)	10(41.7%)	13 (50.0%)	
IgA	12 (24.0%)	6 (25.0%)	6 (23.1%)	
IgD	2 (4.0%)	2 (8.3%)	0 (0.0%)	
Light chain	10 (20.0%)	4 (16.7%)	6 (23.1%)	
Non-secretory	3 (6.0%)	2 (8.3%)	1 (3.8%)	
**RISS stage**				0.028
I	5 (10.0%)	4 (25.0%)	1 (4.3%)	
II	10 (20.0%)	5 (31.3%)	5 (21.8%)	
III	24 (48.0%)	7 (43.7%)	17 (73.9%)	
**DS stage**				0.235
I	1 (2.0%)	1 (6.3%)	0 (0.0%)	
II	0 (0.0%)	0 (0.0%)	0 (0.0%)	
III	38 (76.0%)	15 (93.7%)	23(100.0%)	
**MBD**				< 0.0001
0–2	14 (28.0%)	13 (54.2%)	1(3.8%)	
3–4	36 (72.0%)	11 (45.8%)	25 (96.2%)	
Hemoglobin (g/L) median; range	88 (44–161)	109 (91–161)	69 (44–89)	

*RISS* Revised international staging system,* DS* Durie Salmon,* MBD* myeloma bone disease.

### Cell, reagents, and antibodies

The mouse myeloma cell line 5TGM1 was kindly provided by Dr. Fenghuang Zhan (University of Arkansas for Medical Sciences, Little Rock, USA)^14^. and was cultured in RPMI 1640 (Invitrogen) supplemented with 10% heat-inactivated FBS (Invitrogen), penicillin (100 IU/mL), and streptomycin (100 µg/mL) in a humidified incubator at 37 °C and 5% CO_2_/95% air. CD34^+^ HSPCs were isolated from human BM biopsies with CD34 microbeads (Miltenyi Biotec, Auburn, CA) and cultured in StemSpan SFEM containing Erythroid Expansion Supplement (StemCell Technologies). The following antibodies were purchased from BD Biosciences: CD34-APC (563), CD38-PE-Cy7 (HIT2), CD45RA-FITC (L48) and CD138-PE (MI15). The antibodies CCR1-PE (5F10B29), CCR4-PE (D8SEE), CCR5-PE (NP-6G4) and CD123-PE (6H6) were purchased from eBioscience. Antibodies against p38, phospho-p38, GATA-1, GAPDH, and HRP-conjugated secondary antibodies were purchased from Cell Signaling Technology (CST, USA). CCL3 was purchased from Sigma-Aldrich (St. Louis, MO, USA). The antagonists BX471, AZD2098 and Maraviroc were purchased from MCE (USA).

### Mouse model of MM and in vivo experiments

The spontaneous syngeneic immunocompetent mouse model of MM, C57BL/KaLwRij mice^47^, was purchased from Harlan Laboratories Inc. (Harlan Mice, Netherlands), and the mice were housed under conditions approved by the Ethical Committee for Animal Experiments of Institute of Hematology & Blood Diseases Hospital. Injection of the mouse MM cell line 5TGM1 can accelerate MM development. A total of 1 × 10^6^ 5TGM1 cells suspended in 100 μL of PBS (MM-5TGM1) or the same volume of PBS alone (Con) was injected via tail vein into C57BL/KaLwRij mice. The IgG2b level in the serum and the percentage of 5TGM1 myeloma cells in bone marrow were measured to monitor the tumour burden. Six weeks after tumour cell injection, the mice were euthanized humanely and processed to evaluate erythropoiesis in vivo. Bone marrow was collected and detected by flow cytometry as previously reported^48^.

### Colony formation assays

Bone marrow samples were collected in heparinized tubes and processed with a Ficoll gradient. CD34^+^ cells were isolated using a positive magnetic bead selection protocol (Miltenyi, USA). Purified CD34^+^ cells were seeded in MethoCult complete medium (H4435, StemCell Technologies) following the manufacturer’s instructions. For multilineage colony formation, the cells were plated in 24-well plates in 1 ml of H4435 medium at a density of 1 × 10^3^ cells per well. BM plasma from MM patients was added to MethoCult complete medium at a final concentration of 100 μl/ml. For the antagonist treatment assay, CD34^+^ cells were cultured in medium containing CCL3 (50 ng/ml) and the CCR1 antagonist BX471 (1 μM), the CCR4 antagonist AZD2098 (100 nM), or the CCR5 antagonist maraviroc (15 nM). After 2 weeks of culture, the numbers of colonies (BFU-E, CFU-GM, CFU-GEMM) were counted. BFU-E (burst-forming unit—erythroid) colonies were identified as containing more than 200 erythroblasts in single or multiple clusters and appeared red or brown as the cells contain haemoglobin. CFU-GM (colony-forming unit—granulocyte, macrophage) colonies were identified as containing more than twenty granulocytes and/or macrophages without colour. CFU-GEMM (colony-forming unit—granulocyte, erythroid, macrophage, megakaryocyte) colonies were identified as containing both erythroid cells and twenty or more non-erythroid cells.

### Flow cytometry analysis

Six weeks after tumour cell injection, mice were euthanized humanely. The femurs, tibias, and iliac crest were harvested immediately and placed in PBS. Bone marrow cells were obtained by rinsing femurs, tibias, and iliac crest with PBS. After collection, 1 × 10^6^–5 × 10^6^ cells were suspended in 100 μl of FACS buffer and stained with the appropriate antibodies at 4 °C. According to the manufacturer’s protocol, 10 μl of each antibody was sequentially added to the cell suspension on ice. The workflow is shown in Supplementary Figure 1. Fresh BM cells were stained with the following antibodies: lineage antibodies (B220 (RA3-6B2), CD3 (17A2), Gr-1 (RB6-8C5), CD11b (M1/70), CD8a (53–6.7), CD4 (GK1.5) and Ter-119 (TER-119)), Sca-1-Pe-Cy7 (D7, BD Biosciences), c-Kit-APC (2B8), CD150-PE (TC15-12F12.2), CD48-PerCP (HM48-1, eBioscience), CD41-FITC (MWReg30), CD34-FITC (RAM34, eBioscience), CD16/32-BV421 (93), CD71-FITC (C2, BD Biosciences), CD135-PE (A2F10) and CD138-PE (281-2).

### Cell cycle and apoptosis analysis

For the cell cycle analysis, cells were fixed and permeabilized using a Cytofix/Cytoperm kit (BD Biosciences), stained with anti-Ki-67 antibody, and further incubated with Hoechst 33342 (20 µg/ml). For apoptosis analysis, BM cells were stained for HSCs and then incubated with Annexin V and 7-Amino-Actinomycin D (BD Biosciences). Data were collected on a BD Canto II Flow Cytometer and analysed with FlowJo software (7.6.1).

### RNA extraction and quantitative real-time PCR

Total RNA was isolated using TRIzol reagent (Invitrogen, USA). Aliquots of 1 μg of total RNA were reverse transcribed using an M-MLV reverse transcriptase cDNA synthesis kit (Invitrogen, USA) as described previously^49^. Briefly, the cDNA and appropriate primers were mixed with SYBR Premix Ex Taq (2×) (Takara, China), and qRT-PCR was performed using Quant Studio 5 (Thermo) in a two-step real-time PCR (95 °C for 30 s followed by 40 cycles of 95 °C for 5 s and 60 °C for 30 s). All mRNA levels of target genes were normalized to GAPDH levels and were calculated using the 2^−ΔΔCT^ method. PCR primer sequences were as follows: GATA-1-F: 5′-cctcatccggcccaagaag-3′; GATA-1-R: 5′-atggtcagtggccggttc-3′ KLF-1-F: 5′-tccatcagcacactgaccgc-3′; KLF-1-R: 5′-cggagcgccaccacttga-3′; CCL3-F: 5′-gctgactactttgagacgagc-3′; CCL3-R: 5′-ccagtccatagaagaggtagc-3′; GAPDH-F: 5′-gaaggtgaaggtcggagtc-3′; and GAPDH-R: 5′-gaagatggtgatgggatttc-3′.

### Western blotting

Western blotting was performed as previously described^50^. Briefly, protein samples were separated using SDS-PAGE and transferred onto PVDF membranes. The membranes were blocked with 5% non-fat dry milk in TRIS-buffered saline (TBS) containing 0.05% Tween-20 (TBST) prior to incubation overnight at 4 °C with primary antibody against phosphorylated p38, total p38 or GATA1 (1:1000 dilution) overnight at 4 °C. The appropriate HRP-conjugated secondary antibodies were added, and protein signals were developed with the use of enhanced chemiluminescence (ECL) reagents (Thermo Scientific). The developed images were obtained and analysed using ChemiDoc (Bio-Rad, USA).

### Immunofluorescence analysis

Immunofluorescence was performed as previously described^48^. HSPCs were spun down on glass slides and then fixed in − 20 °C acetone/methanol (1:1 volume) for 10 min. Cells were blocked by immersion in 5% goat serum for 60 min. Cells were then incubated with primary antibody against phosphorylated p38 or GATA1 (1:100 dilution in PBS containing 4% bovine serum albumin, CST) overnight at 4 °C and incubated with the secondary antibodies Alexa Fluor 488-conjugated goat anti-mouse immunoglobulin G (IgG; H + L; 1:200, Invitrogen) or Alexa Fluor 568-conjugated goat anti-rabbit IgG at room temperature for 30 min in the dark. Nuclei were stained with DAPI. The slides were sealed with nail polish. Microscopic images were captured using an Ultra VIEW Vox confocal microscope (Perkin Elmer, USA).

### ELISA assays

Six weeks after tumour cell or PBS injection, BM plasma and serum from peripheral blood (PB) were collected from each mouse. ELISA for CCL3 (R&D Systems, Minneapolis, MN, USA) was performed on the serum samples and BM supernatants. A mouse IgG2b ELISA Quantitation Set (Bethyl Laboratories) and ELISA Starter Accessory Kit (Bethyl Laboratories) were used to determine Ig secretion according to the manufacturer’s instructions. The results were obtained by measuring the absorbance (450 nm) with a microtiter plate reader, and the Ig light chain content was determined using Curve Expert (1.4) software.

### RNA-seq and GSEA analysis

CD34^+^ cells from the bone marrow of myeloma patients and healthy donors were collected using CD34 microbeads. RNA was extracted using the Qiagen RNeasy Kit. Library construction and RNA sequencing were performed on a BGISEQ-1000 system by the Beijing Genomic Institution (www.genomics.org.cn, BGI, Shenzhen, China). Gene set enrichment analysis (GSEA) was performed with GSEA_4.0.3 (gene set enrichment analysis, Broad Institute).

### Statistical analyses

Statistical analyses were performed using IBM SPSS 19.0 software and GraphPad Prism 6.0. Unpaired Student’s *t*-test was used to generate p values for most of the data sets. Values are represented as the mean ± standard error of the mean (SEM) values from at least three separate experiments; *p < 0.05, **p < 0.01, ***p < 0.001, and ****p < 0.0001. Survival analysis was performed by Kaplan–Meier analysis.

## Supplementary information

Supplementary Figures.

Supplementary Legends.

## Data Availability

The data sets used and/or analysed in the current study are available from the corresponding author upon reasonable request.
