# Supplementary material for: Multiple myeloma hinders erythropoiesis and causes anaemia owing to high levels of CCL3 in the bone marrow microenvironment
Source: Sci Rep. 2020 Nov 25;10:20508. doi: 10.1038/s41598-020-77450-y (PMC7689499; doi:10.1038/s41598-020-77450-y)
Supplement: Supplementary file 1 — Supplementary Figures. [file 41598_2020_77450_MOESM1_ESM.pptx]

## Slide 1
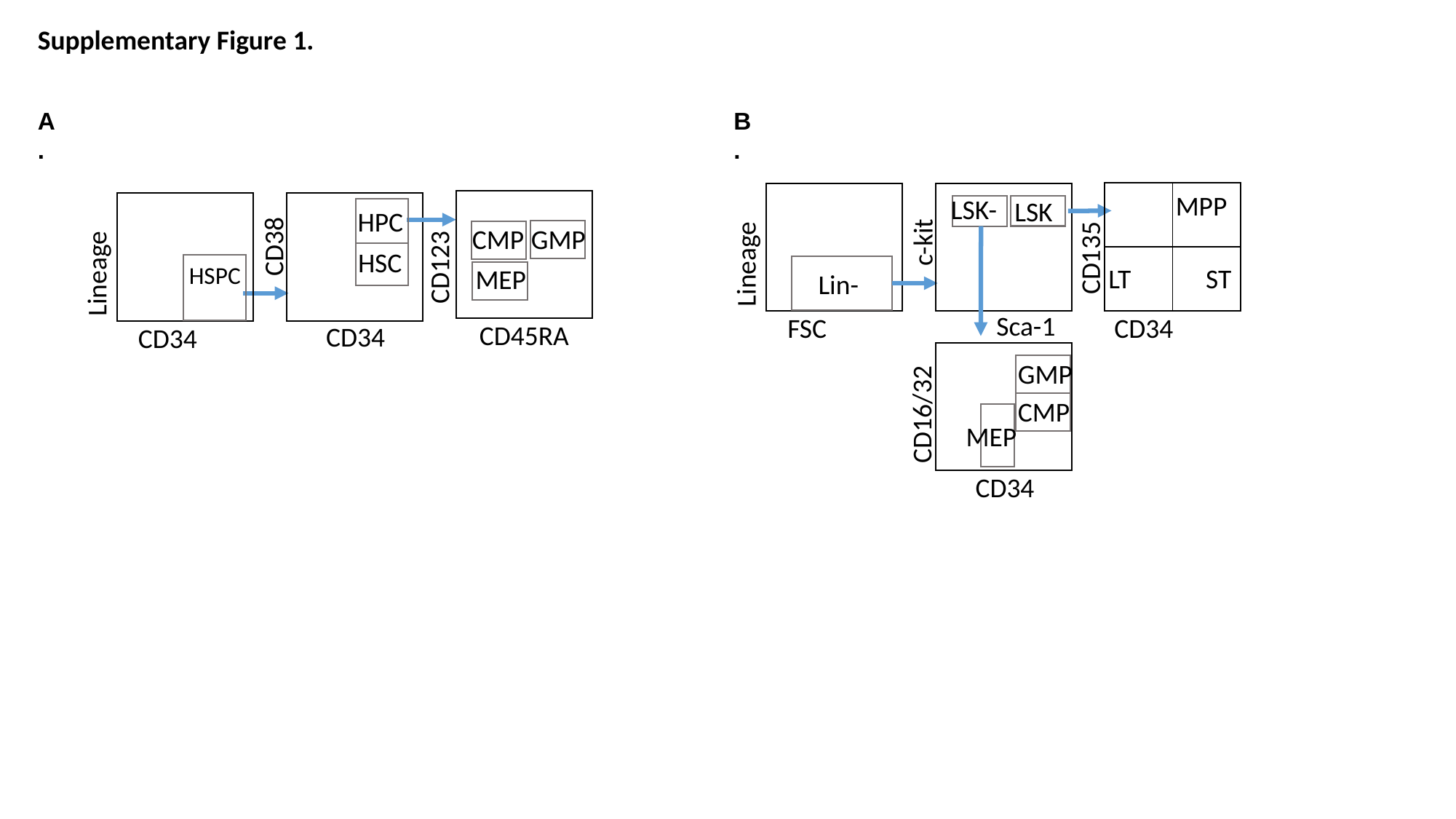

Supplementary Figure 1.
B.
A.
MPP
LSK-
LSK
c-kit
HPC
CD38
CMP
GMP
CD135
CD123
Lineage
HSC
Lineage
HSPC
ST
LT
MEP
Lin-
Sca-1
CD34
FSC
CD45RA
CD34
CD34
GMP
CMP
CD16/32
MEP
CD34

## Slide 2
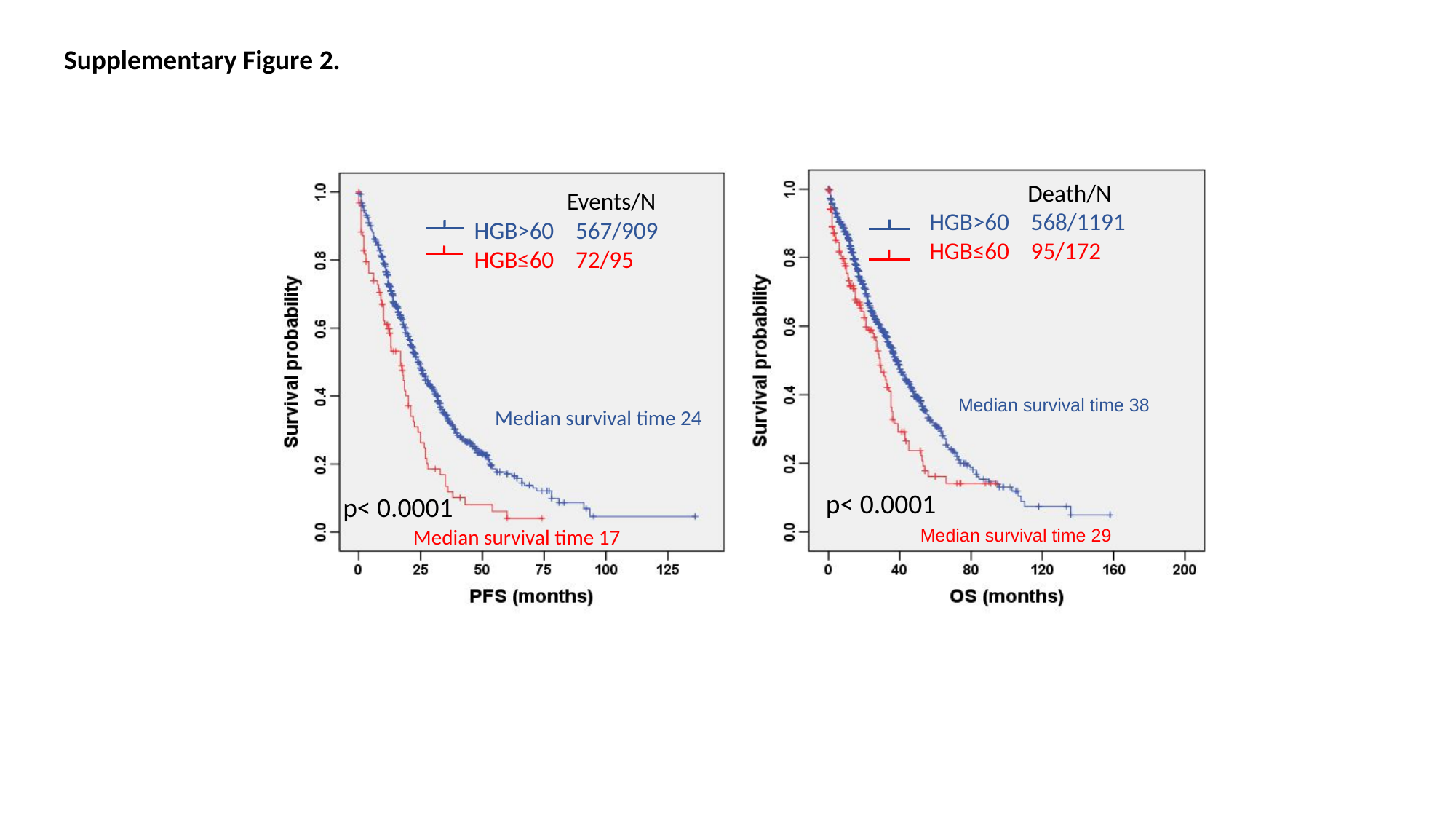

Supplementary Figure 2.
 Events/N
HGB>60 567/909
HGB≤60 72/95
Median survival time 24
p< 0.0001
Median survival time 17
 Death/N
HGB>60 568/1191
HGB≤60 95/172
Median survival time 38
p< 0.0001
Median survival time 29

## Slide 3
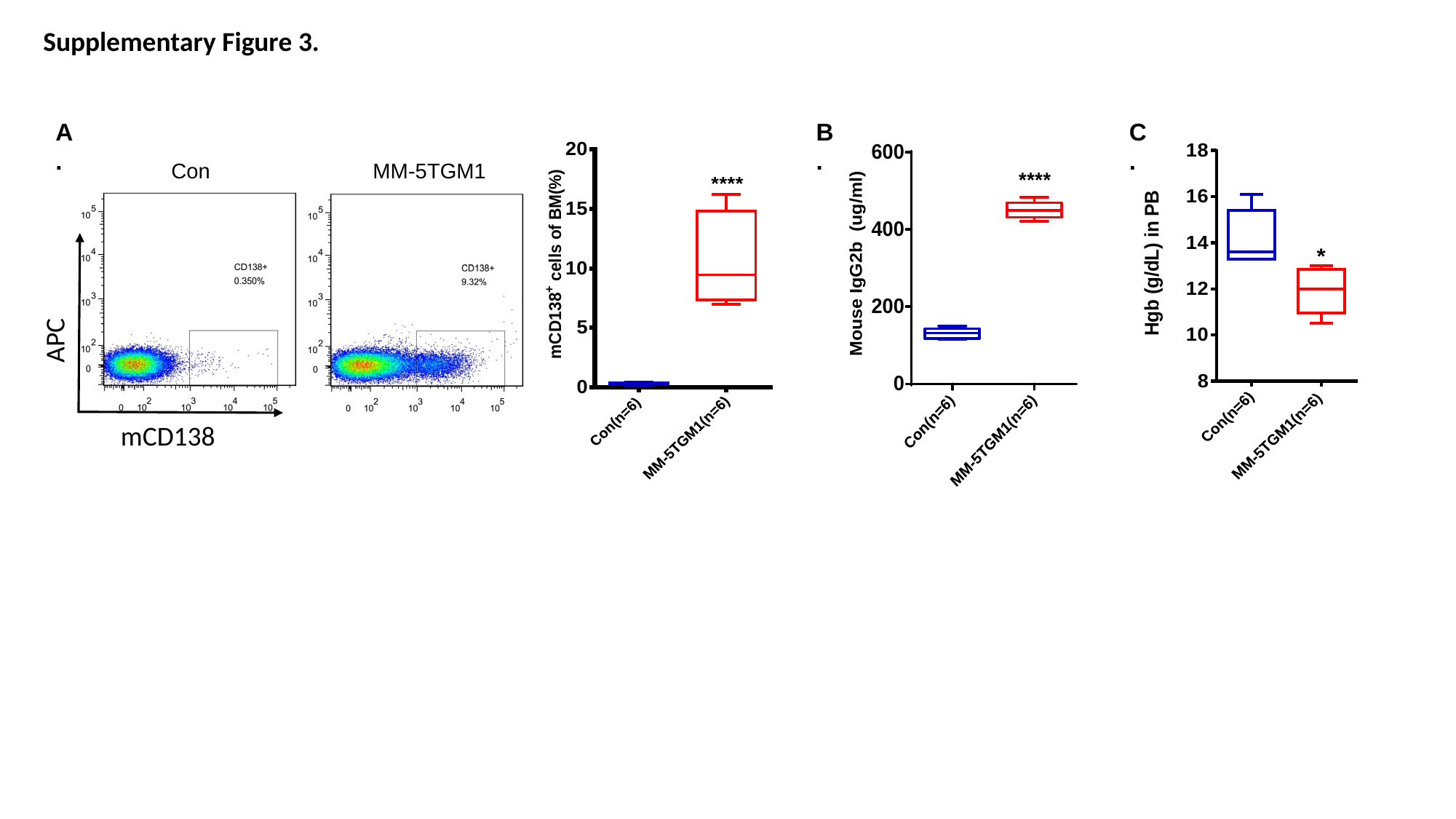

Supplementary Figure 3.
A.
B.
C.
MM-5TGM1
Con
APC
mCD138

## Slide 4
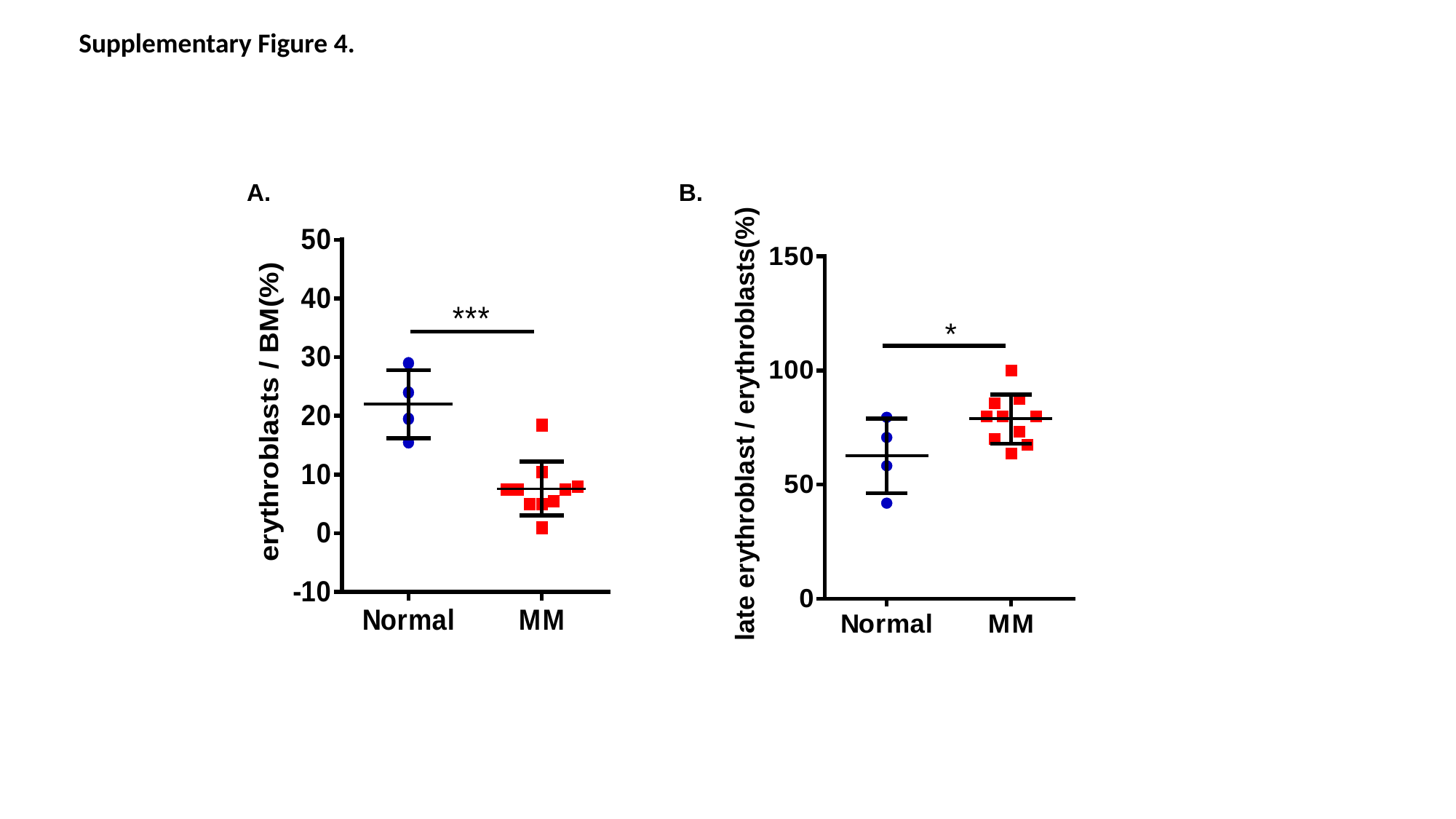

Supplementary Figure 4.
A.
B.

## Slide 5
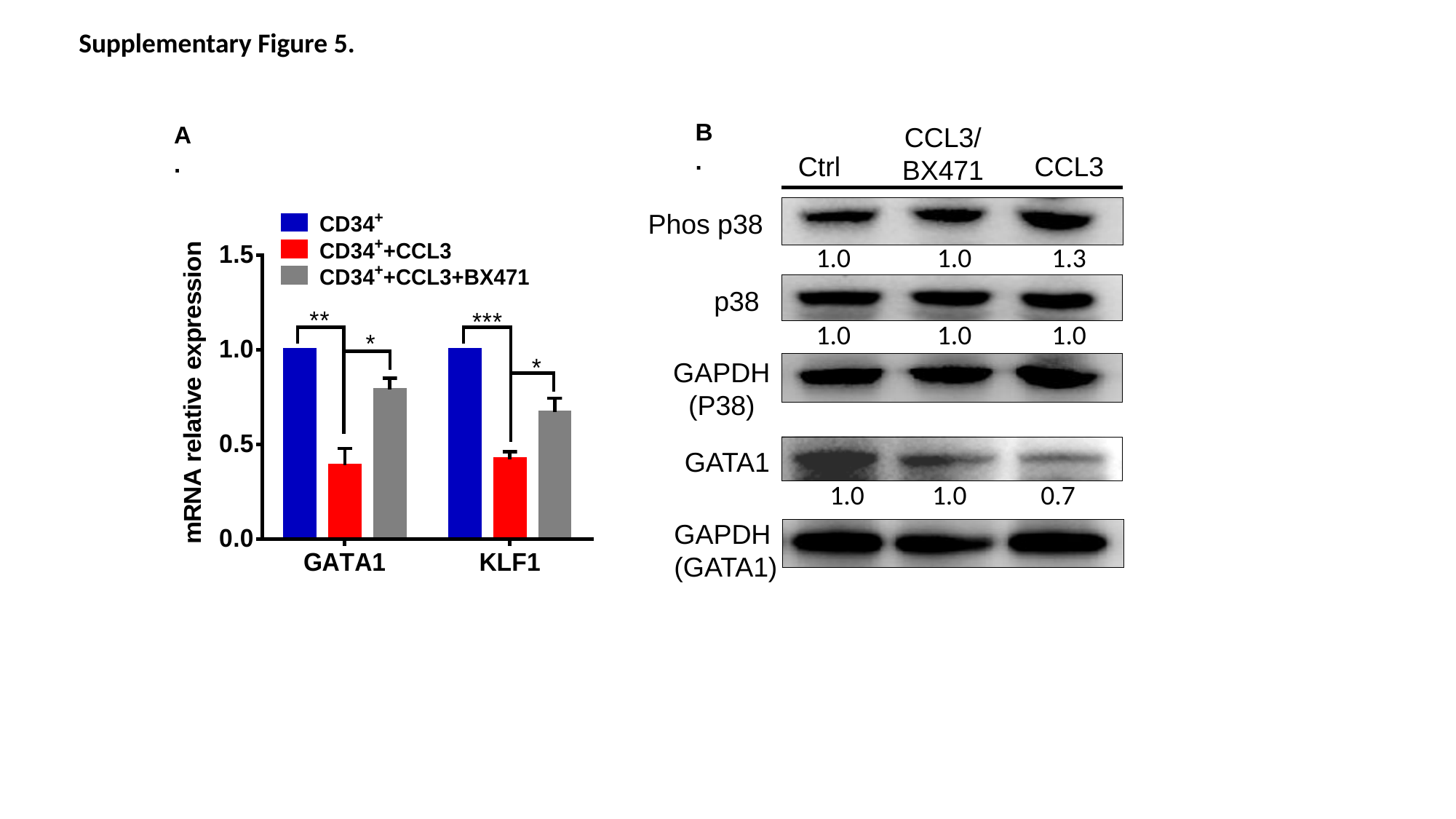

Supplementary Figure 5.
B.
A.
CCL3/ BX471
Ctrl
CCL3
Phos p38
1.0 1.0 1.3
 p38
1.0 1.0 1.0
GAPDH
 (P38)
GATA1
1.0 1.0 0.7
GAPDH
(GATA1)

## Slide 6
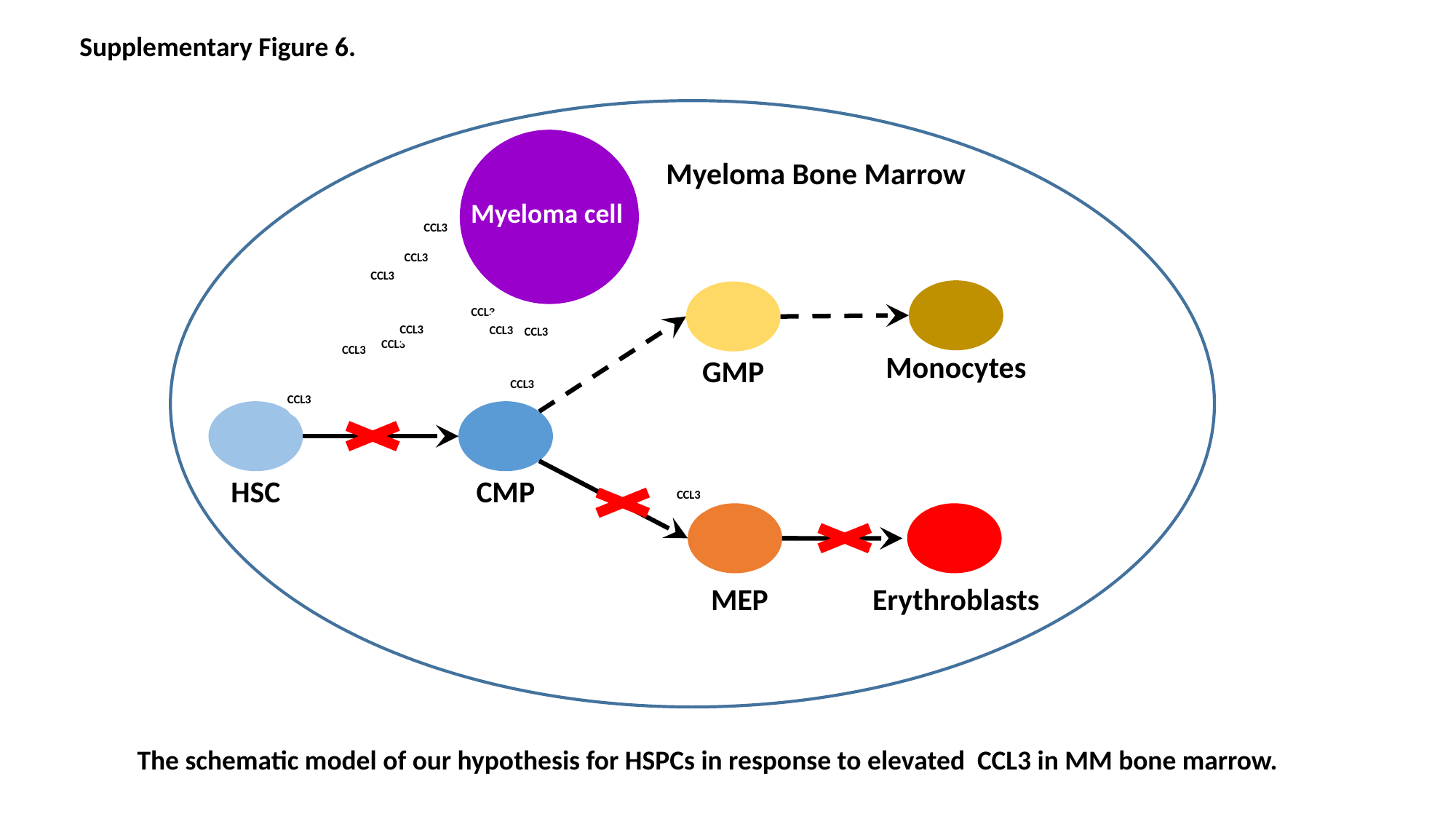

Supplementary Figure 6.
Myeloma cell
Myeloma Bone Marrow
CCL3
CCL3
 CCL3
 CCL3
 CCL3
 CCL3
 CCL3
 CCL3
 CCL3
Monocytes
GMP
 CCL3
 CCL3
 CCL3
HSC
CMP
MEP
Erythroblasts
The schematic model of our hypothesis for HSPCs in response to elevated CCL3 in MM bone marrow.
