# Supplementary material for: Multiple myeloma hinders erythropoiesis and causes anaemia owing to high levels of CCL3 in the bone marrow microenvironment
Source: Sci Rep. 2020 Nov 25;10:20508. doi: 10.1038/s41598-020-77450-y (PMC7689499; doi:10.1038/s41598-020-77450-y)
Supplement: Supplementary file 2 — Supplementary Legends. [file 41598_2020_77450_MOESM2_ESM.docx]

**Multiple Myeloma Hinders Erythropoiesis and Causes Anaemia Owing to High Levels of CCL3 in the Bone Marrow Microenvironment**

Lanting Liu^1#^, Zhen Yu^1#^, Hui Cheng^1^, Xuehan Mao^1^, Weiwei Sui^1^, Shuhui Deng^1^, Xiaojing Wei^1^, Junqiang Lv^2^, Chenxing Du^1^, Jie Xu^1^, Wenyang Huang^1^, Shuang Xia^3^, Gang An^1^, Wen Zhou^4^, Xiaoke Ma^5^*, Tao Cheng^1^, Lugui Qiu^1^, Mu Hao^1^*

1. State Key Laboratory of Experimental Hematology, National Clinical Research Center for Blood Diseases, Institute of Hematology & Blood Diseases Hospital, Chinese Academy of Medical Sciences & Peking Union Medical College, Tianjin 300020, China;

2. Department of Immunology, Key Laboratory of Immune Microenvironment and Disease of the Educational Ministry of China, Tianjin Key Laboratory of Cellular and Molecular Immunology, School of Basic Medical Sciences, Tianjin Medical University, 300070, Tianjin, China;

3. Department of Radiology, Tianjin First Central Hospital, Tianjin, 300192, China；

4. Cancer Research Institute, Key Laboratory of Carcinogenesis and Cancer Invasion, Ministry of Education; Key Laboratory of Carcinogenesis, National Health and Family Planning Commission, Central South University, Hunan, China;

5. School of Computer Science and Technology, Xidian University, Xi’an, China.

* Correspondence: [haomu@ihcams.ac.cn](mailto:haomu@ihcams.ac.cn) & xkma@xidian.edu.cn

# The authors contribute equally to this work.

**Supplementary Appendix**

**Supplemental Figure 1.**

(A) Flow chart of flow cytometry analysis of human HSPCs. (B) Flow chart of flow cytometry analysis of mouse HSPCs.

**Supplemental Figure 2.**

Kaplan-Meier analysis of PFS and OS for NDMM patients according to the levels of hemoglobin.

**Supplemental Figure 3.**

(A) Flow plots (left) and histogram (right) show the percentage of mCD138^+^ cells in bone marrow from MM-5TGM1 mice and sex/age-matched Con-5TGM1 mice. (B) The concentrations of immunoglobulin light chain proteins in the serum of different groups were determined with ELISA. (C) Hemoglobin in the PB of MM-5TGM1 mice and sex/age-matched Con-5TGM1 mice.

**Supplemental Figure 4.**

(A) The clinical data showed the proportion of erythroblasts in BM from normal donors (n=4) and NDMM patients (n=10). (B) The proportion of late erythroblast in erythroblasts from normal donors (n=4) and NDMM patients (n=10).

**Supplemental Figure 5.**

(A) RT-PCR analysis showed the level of GATA1 and KLF1 in CD34^+^ cells from healthy control in the presence of CCL3/BX471 or not. GAPDH was used as an internal control. (B) Western blotting analysis of phosphorylated p38 (phos p38), total p38 and GATA1 after the induction of erythroid differentiation in the presence of CCL3/BX471 or not. GAPDH used as an internal control.

**Supplemental Figure 6.**

The schematic model of our hypothesis for HSPCs in response to elevated CCL3 in MM
